# Aicardi-Goutières syndrome-associated mutation at ADAR1 gene locus activates innate immune response in mouse brain

**DOI:** 10.1186/s12974-021-02217-9

**Published:** 2021-07-31

**Authors:** Xinfeng Guo, Clayton A. Wiley, Richard A. Steinman, Yi Sheng, Beihong Ji, Junmei Wang, Liyong Zhang, Tony Wang, Mazen Zenatai, Timothy R. Billiar, Qingde Wang

**Affiliations:** 1 Department of Surgery, University of Pittsburgh School of Medicine, 200 Lothrop Street, Pittsburgh, PA BSTW943 USA; 2 Department of Pathology, University of Pittsburgh School of Medicine, Pittsburgh, PA USA; 3 Department of Medicine, University of Pittsburgh School of Medicine, Pittsburgh, PA USA; 4 Magee Women Research Institute, University of Pittsburgh, Pittsburgh, PA USA; 5 Department of Pharmaceutical Sciences and Computational Chemical Genomics Screening Center, University of Pittsburgh School of Pharmacy, Pittsburgh, PA USA; 6 Division of Viral Products, Center for Biologics Evaluation and Research, U.S. Food and Drug Administration, Silver Spring, MD 20993 USA; 7 V.A. Pittsburgh Health System, Pittsburgh, PA USA; 8 Pittsburgh Liver Research Center, University of Pittsburgh Medical Center, University of Pittsburgh School of Medicine, Pittsburgh, PA USA

**Keywords:** Aicardi-Goutières syndrome (AGS), Animal model, RNA editing, Adenosine Deaminase Acting on RNA 1 (ADAR1), Interferonopathy, In situ hybridization

## Abstract

**Background:**

Aicardi-Goutières syndrome (AGS) is a severe infant or juvenile-onset autoimmune disease characterized by inflammatory encephalopathy with an elevated type 1 interferon-stimulated gene (ISG) expression signature in the brain. Mutations in seven different protein-coding genes, all linked to DNA/RNA metabolism or sensing, have been identified in AGS patients, but none of them has been demonstrated to activate the IFN pathway in the brain of an animal. The molecular mechanism of inflammatory encephalopathy in AGS has not been well defined. Adenosine Deaminase Acting on RNA 1 (ADAR1) is one of the AGS-associated genes. It carries out A-to-I RNA editing that converts adenosine to inosine at double-stranded RNA regions. Whether an AGS-associated mutation in ADAR1 activates the IFN pathway and causes autoimmune pathogenesis in the brain is yet to be determined.

**Methods:**

Mutations in the ADAR1 gene found in AGS patients were introduced into the mouse genome via CRISPR/Cas9 technology. Molecular activities of the specific p.K999N mutation were investigated by measuring the RNA editing levels in brain mRNA substrates of ADAR1 through RNA sequencing analysis. IFN pathway activation in the brain was assessed by measuring ISG expression at the mRNA and protein level through real-time RT-PCR and Luminex assays, respectively. The locations in the brain and neural cell types that express ISGs were determined by RNA in situ hybridization (ISH). Potential AGS-related brain morphologic changes were assessed with immunohistological analysis. Von Kossa and Luxol Fast Blue staining was performed on brain tissue to assess calcification and myelin, respectively.

**Results:**

Mice bearing the ADAR1 p.K999N were viable though smaller than wild type sibs. RNA sequencing analysis of neuron-specific RNA substrates revealed altered RNA editing activities of the mutant ADAR1 protein. Mutant mice exhibited dramatically elevated levels of multiple ISGs within the brain. RNA ISH of brain sections showed selective activation of ISG expression in neurons and microglia in a patchy pattern. ISG-15 mRNA was upregulated in ADAR1 mutant brain neurons whereas CXCL10 mRNA was elevated in adjacent astroglia. No calcification or gliosis was detected in the mutant brain.

**Conclusions:**

We demonstrated that an AGS-associated mutation in ADAR1, specifically the p.K999N mutation, activates the IFN pathway in the mouse brain. The ADAR1 p.K999N mutant mouse replicates aspects of the brain interferonopathy of AGS. Neurons and microglia express different ISGs. Basal ganglia calcification and leukodystrophy seen in AGS patients were not observed in K999N mutant mice, indicating that development of the full clinical phenotype may need an additional stimulus besides AGS mutations. This mutant mouse presents a robust tool for the investigation of AGS and neuroinflammatory diseases including the modeling of potential “second hits” that enable severe phenotypes of clinically variable diseases.

**Supplementary Information:**

The online version contains supplementary material available at 10.1186/s12974-021-02217-9.

## Background

Aicardi-Goutières syndrome (AGS) refers to a group of genetic diseases characterized by severe inflammatory encephalopathy that usually presents within the first year of life, resulting in progressive loss of cognition, intellectual regression, spasticity, dystonia, and motor disability [1–3]. There currently exists no effective therapy for this disorder. Few patients survive past childhood, although some individuals who develop the condition later or have milder neuropathology may live into adulthood [4–6]. AGS was first reported as a progressive familial encephalopathy in infancy with calcifications of the basal ganglia and chronic cerebrospinal fluid lymphocytosis [1]. Elevated type 1 interferon (IFN) and IFN-stimulated gene (ISG) expression were found in the central nervous system (CNS) and in peripheral blood cells of AGS patients [7–9]. Pedigree analysis indicated that AGS had a genetic basis; however, it was not until two decades later that mutations in any specific genes—namely, TREX1 and RNase H2—were implicated in AGS pathogenesis [10, 11]. Since then, mutations in seven protein-coding genes have been linked to AGS, i.e., TREX1, RNASEH2A, RNASEH2B, RNASEH2C, SAMHD1, ADAR1, and IFIH1 [3, 6]. All of these AGS-linked genes encode proteins involved in nucleotide metabolism and/or signaling of DNA/RNA sensing that trigger type I IFN signaling activation [12].

Knockdown of TREX1, SAMHD1, RNASEH2A, and ADAR1 expression in astrocytes and brain endothelial cells led to increased release of proinflammatory cytokines in vitro [13]. Knockout (KO) and mutant mouse models of TREX1 [14–18], and ADAR1 [19–21] manifest increased type 1 interferon signaling and peripheral autoimmunity [22]. These findings support a connection between dysregulated nucleic acid metabolism, activation of DNA/RNA sensing signaling, and type I IFN-induced tissue injury in AGS pathogenesis [8]. However, the molecular mechanism of encephalopathy in AGS remains to be elucidated, and the link between the genetic mutations and brain inflammatory injury has not been established. There is substantial clinical heterogeneity in patients carrying the same AGS-linked mutations [9, 23, 24]. In some cases, the onset of disease was preceded by viral infection or vaccination [9]. In animal models, none of the AGS-associated mutations has been demonstrated to cause an inflammatory or pathogenic phenotype in the brain. This raises a question of the sufficiency of a specific AGS-associated mutation to activate the INF signaling pathway in the brain, the hallmark of AGS pathogenesis.

Adar1, one of the AGS-linked genes, encodes an RNA editing enzyme that converts adenosine to inosine in double-stranded RNAs [25]. The catalytic activity of ADAR1 prevents activation of the IFN pathway by endogenous double-stranded RNA [20]. Adar1 knockout in mice results in embryonic death at E11.5 to E12.5 days as shown in various mouse models [20, 21, 26, 27]. Even a single nucleotide mutation in the catalytic domain of ADAR1 [20] or a partial deletion of its RNA editing-unrelated N-terminus [28] is embryonic lethal. Type I IFN and ISG expression are significantly elevated in the embryonic cells from ADAR1 KO mice [19, 20]. Deletion of Adar1 in adult tissues in conditional KO models also causes cell death and tissue injury due to activation of IFN pathways [26, 29, 30]. We and others have demonstrated a role for ADAR1 in limiting RNA sensing and downstream interferon activation [31]. ADAR1 inhibits the innate immune RNA sensor MDA5, and knockout of MDA5 rescues ADAR1 knockouts from lethality [20, 21, 32]. However, no animal model has been produced that enables testing of whether mutation of Adar1 activates the IFN pathway and causes inflammatory injury in the brain.

Here, we report a successful establishment of a mouse model in which a known AGS missense mutation in the ADAR1 gene was introduced into the mouse genome, precipitating the aspect of interferonopathy in the brain.

## Materials and methods

### Mouse genome mutagenesis

Mouse genome mutagenesis was carried out through the CRISPR/Cas9 gene editing approach in one-cell-stage embryos. A mixture of Cas9 protein 100 ng/μl (Alt. R S.p. Cas9 Nuclease 3NLS IDT Cat # 1,074,181), single guide RNAs (sgRNA) 200 ng/μl, and ssODN (DNA template) 200 ng/μl was transfected into the embryos through electroporation using the Super Electroporator NEPA21 type II and CUY 501–1-1.5 electrode (NEPA GENE Co. Ltd, Chiba, Japan). According to the original reported mutation sites in the Adar1 gene found in AGS patients [33], we designed 8 sets of sgRNAs and mutant oligos to target the mouse genome at the genetic positions equivalent to each of the missense mutations of AGS patients (Supplemental Fig. 1). The sgRNAs were synthesized by in vitro transcription (MAXIscript™ T7 Transcription Kit, Thermo Fisher) from PCR products harboring the guide RNA sequences. Then, the sgRNAs were purified using an RNA Cleanup kit (Qiagen), according to the manufacturer’s instructions. The ssODN oligos were synthesized by Integrated DNA Technologies (IDT), with each of the ssODN carrying one of the ADAR1 mutations. The electroporated embryos were washed two times in KSOM medium, then maintained in KSOM medium overnight at 5% CO_2_ at 37 °C. The following day, the two-cell-stage embryos were transferred to the oviducts of pseudopregnant CD1 females (0.5 dpc). The potential founders carrying the designed mutation at the targeted ADAR1 loci were screened among the living pups. Multiple trials were performed for each mutation site. Sanger sequencing was used for genome sequence screening after PCR amplification of the targeted gene regions. In this study, we only succeeded with two of the designed mutations (see the “Results” section), the c.2844 G > T mutation (equivalent to c.2997 G > T in patients), encoding the change for p.K948N in mouse ADAR1 protein, which is equivalent to the p.K999N mutation found in AGS patients, and the c. 583 C > G mutation (c.577 C > G in patients), encoding the change for p.P195A, equivalent to the human p.P193A mutation [9, 33]. For the K948N mutation, the sgRNA sequence was 5′-gcaaggcaagcttcgcacca-3′. The ssODN sequence for the G to T mutation at 2997 of the ADAR1 gene was 5′-gtgccgtggaaagcacagagtcccgccattaccctgtctttgaaaatcccaagcaaggcaatcttcgcaccaaagtggagaatggtgagtggtaggtgccagctggcagtgaggagacatgcacgcgaggggtgtccgcttcctt-3′. The primer sequences used for amplifying the region flanking K999N mutation were 5′-tgccagttcccacataggat-3′ and 5′-agtccagtgacacccacctc-3′. For P195A mutation, the sgRNA sequence was 5′-gcaaggcaagcttcgcacca-3′. The ssODN sequence for the G to T mutation at 583 of the Adar1 gene was 5′-gtgccgtggaaagcacagagtcccgccattaccctgtctttgaaaatcccaagcaaggcaatcttcgcaccaaagtggagaatggtgagtggtaggtgccagctggcagtgaggagacatgcacgcgaggggtgtccgcttcctt-3′. The primer sequences used for amplifying the region flanking K999N mutation were 5′-tgccagttcccacataggat-3′ and 5′-agtccagtgacacccacctc-3′.

Founders carrying the designed mutations were bred to homozygosity for phenotypic analysis.

### Genotyping analysis

PCR genotyping approaches were established for the two mutant mouse lines. For K948N mutation, the sequences of the primers are 5′-aaaatcccaagcaaggcaag-3′ and 5′-gctgtgtggtgactgcattt-3′ for the wild type allele and 5′-cacactgccaagaacagcat-3′ and 5′-ctccactttggtgcgaaga-3′ for the mutant allele. PCR conditions were 94 °C 4 min, 94 °C 30 s, 63 °C 30 s, and 72 °C 30 s for 30 cycles. For P195A mutation, the sequences of the primers are 5′-aaaatcccaagcaaggcaag-3′ and 5′-gctgtgtggtgactgcattt-3′ for the wild type allele and 5′-cacactgccaagaacagcat-3′ and 5′-ctccactttggtgcgaaga-3′ for the mutant allele. PCR conditions were 94 °C 4 min, 94 °C 30 s, 63 °C 30 s, and 72 °C 30 s for 30 cycles. These PCR conditions were optimized to identify the single nucleotide replacements and distinguish the mutation from the wild type gene alleles.

### Mouse breeding and phenotype observation

Mice were hosted in a SPF animal facility in the University of Pittsburgh School of Medicine with strict monitoring of temperature, humidity, light cycles, and potential presence of pathogens. Studies were approved by IACUC at the University of Pittsburgh. The mice were observed from birth to adulthood for growth, behavioral change, and signs of neuropathy. The body weights of the mice, together with their littermates, were measured weekly. PCR genotyping was performed on each of the mice at 2–3 weeks of age to determine their genetic status.

### Protein structure modeling and comparison

Mouse and human ADAR1 protein sequences, Q99MU3 and P55265 in UniPro protein database, were used for protein modeling and structure comparison. The protein structure modeling software package Modeller [34] was used for the protein structure prediction; human ADAR2 crystal structure (PDB Code 1ZY7) was used as the template for the modeling. 22 amino acids, AA922-943 in mouse ADAR1 sequence, were skipped as the corresponding structure is not found in the template. The molecular dynamics simulation software package AMBER (*AMBER 2018. University of California, San Francisco 2018*) was used for the structural refinement. Modeling was conducted both for mouse and for human sequences.

### Pathology study

Histopathologic studies were carried out on formalin-fixed paraffin-embedded (FFPE) mouse tissues including the brain, skin, heart, lung, liver, spleen, and kidney. Tissues were harvested and immersion fixed in 4% paraformaldehyde in phosphate-buffered saline followed by dehydration, paraffin embedding, and routine pathologic processing. Hematoxylin and eosin (H&E), Von Kossa, and Luxol Fast Blue staining were performed on tissue sections.

### Immunohistochemistry

Five-micrometer-thick sections from paraffin-embedded brain blocks were immunohistochemically stained for glial fibrillary acidic protein (GFAP) and ionized calcium binding adaptor molecule 1 (Iba1) with antibodies mouse anti-GFAP (catalog# 837,202, BioLegend) and rabbit anti-IBA-1 (catalog# WDG5619, WAKO) each at a dilution of 1:1000, followed by secondary antibodies and peroxidase development [35].

### RNA in situ hybridization

ISH studies were performed on FFPE tissue sections using 2 commercial RNAscope Target Probes (Advanced Cell Diagnostics, Hayward, CA) catalog # 559,271 and 408,921 complementary to sequences 2–561 of ISG-15 and 11–1012 of CXCL10, respectively. Pretreatment, hybridization, and detection techniques (RNAscope 2.5HD) were performed according to the manufacturer’s protocols and as previously described [36].

### Protein sample preparation and analysis

Before tissues were collected from the mice, whole body perfusion with PBS with heparin was performed to remove the blood from the organs. The tissues were immediately frozen in liquid nitrogen until analysis. For the brain sample collections, the cerebellum and olfactory bulb were removed prior to freezing. Homogenization was performed in RIPA buffer with the addition of protease inhibitor cocktail. ADAR1 protein in the brain tissues was detected by Western blot as described previously [31]. In brief, 30 μg of protein extract was loaded to each lane and separated on 8% polyacrylamide gel with 0.1% SDS. ADAR1 was detected with ADAR1 antibody clone 15.8.6 (Santa Cruz sc-73408) at 1:1000 dilution.

### RNA sample preparation and qRT-PCR

RNA isolation was performed with RNeasy Plus Mini Kit (Qiagen Cat # 74,134) following the manufacturer’s instructions. Quantitative RT-PCR was performed using the iTaq™ Universal SYBR Green One-Sep Kit (Bio-Rad cat #1,725,151). Assayed genes comprised ISG15, Ccl-5, Ccl-10, Ifit-1, Ifit3, Oasl-1, Oasl-2, Mx2, IL-6, TNF α, Xaf1, IFI 27, Oas1c, IL-1, IFN-α, IFN-β, GAPDH, and HPRT. Primer sequences are listed in the Supplemental material. The specificity of PCR amplifications was confirmed by the melting curve and by electrophoresis analysis of the final PCR products. The quantification of the mRNA levels was calculated by the Ct values using the ΔΔt method with internal references of the average value of the HPRT and GADH expressions.

### Cytokine and chemokine assays

Forty-five [45] cytokines and chemokines were measured utilizing Luminex® xMAP® technology. The multiplexing analysis was performed using the Luminex™ 200 system (Luminex, Austin, TX, USA) by Eve Technologies Corp. (Calgary, Alberta, Canada). Forty-five markers were simultaneously measured in the samples using a MILLIPLEX Mouse Cytokine/Chemokine 32-plex kit and a MILLIPLEX Mouse Cytokine/Chemokine 13-plex kit (Millipore, St. Charles, MO, USA) according to the manufacturer’s protocol. The 45-plex consisted of Eotaxin, Erythropoietin, 6Ckine, Fractalkine, G-CSF, GM-CSF, IFNB1, IFNγ, IL-1α, IL-1β, IL-2, IL-3, IL-4, IL-5, IL-6, IL-7, IL-9, IL-10, IL-11, IL-12 (p40), IL-12 (p70), IL-13, IL-15, IL-16, IL-17, IL-20, IP-10, KC, LIF, LIX, MCP-1, MCP-5, M-CSF, MDC, MIG, MIP-1α, MIP-1β, MIP-2, MIP-3α, MIP-3B, RANTES, TARC, TIMP-1, TNFα, and VEGF. The assay sensitivities of these markers range from 0.3 to 30.6 pg/mL.

### Liver function assays

Mouse blood was taken via cardiopuncture and serum was collected and frozen at − 80 °C until analysis. ALT, AST, and total bilirubin were measured at the central laboratory at UPMC pathology core.

### RNA editing essays

Total brain RNA was isolated from undissected whole brains, and reverse transcript PCR was performed with the total RNA samples. The PCR products of the entire brain RNA pool were subjected to Sanger sequencing analysis. The relative quantities of inosine (read as guanosine) and adenosine at each editing site were determined on the chromatographs by the ratio of the G and A peaks. The primer sequences used for the PCR amplifications of the editing sites were 5′-cactgaggaatttgaagatgga-3′ and 5′-agcaggcatggaatgatagg-3′ (for the GRIA2 Q/R site and the intron hot spots), 5′-cttgcgacaccatgaaagtg-3′ and 5′-gccagaaatgtgggtaaagg-3′ (for the GRIA2 R/G site), 5′-agcagagaaagccgtgtgat-3′ and 5′-agaacaccacatccatgcaa-3′ (for the GRIA3 R/G site), 5′-acccgtgcaaccctgact-3′ and 5′-ttgcaggaaattttgtccagt-3′ (for the GRIK1 Q/R site), and 5′-attatgtctggcctttacctagatat-3′ and 5′-ataggaactgaaactcctattgatattgc-3′ (for the editing sites A to E in 5-HT 2cR mRNA). RNA editing efficiency was assessed on the editing sites in GRIA2, GRIA3, and GRIK1 mRNAs for the Q/R and R/G sites and on the A-E sites in 5-HT2C receptor mRNA by calculation of the relative ratio of the average of G peak to A peak on each of the Sanger sequencing chromatograph for samples from mutant and wild type groups.

### Data analysis

Continuous data was summarized using median and interquartile or mean and standard deviation. For comparison between two groups of nonparametric data, the Wilcoxon rank-sum test was used to test differences between two independent groups. To test the differences between more than two groups non-parametrically, Kruskal–Wallis test by ranks was used and followed by the post hoc Conover test for pairwise multiple comparisons procedure upon the rejecting the null of the Kruskal–Wallis test. All tests used in the analysis were of two-sided nature with *p* ≤ 0.05 was referred as statistically. Prism-GraphPad was used to generate graphs and Stata software version 12.0 (StataCorp, College Station, TX) was used in conducting statistical testing.

## Results

### Successful introduction of ADAR1 missense mutations into the mouse genome

Nine AGS-linked mutations in the human ADAR1 gene, specifically eight missense and one frameshift mutations, were originally reported [33], with additional data from 37 affected families (9) identifying a total of 28 ADAR1 mutations (12 missense, 16 nonsense). To test whether an ADAR1 mutation could recapitulate AGS pathogenesis and to establish an animal model for the future study of AGS, we sought to introduce all the missense mutations reported in the original paper [33] into the mouse genome. CRISPR/Cas9 technology was used for our study, and the single guide RNAs (sgRNA) and mutant DNA templates were designed to make single nucleotide replacements at every corresponding locus. However, only wild type progeny arose for six of the mutations in multiple trials, consistent with the embryonic lethality of ADAR1 gene interruptions [20, 21, 26, 27]. Ultimately, 2 of the 8 designed mutant strains were produced after screening 108 living pups from eleven independent experiments for these two specific mutation sites (Supplemental Fig 1). The first strain carried the c.2844 G > T single nucleotide replacement, encoding the change for p.K948N in mouse ADAR1 in the catalytic domain, which is highly conserved between human and mouse. This K948N mutation is equivalent to the K999N mutation in human. The sequences flanking this mutation site are almost identical in these two species (Supplemental Fig 2), indicating a same or similar effect of this mutation in human and mouse. The second strain carried the c.583 C > G mutation (c.577 C > G in human), encoding a change of p.P195A in the N-terminus of ADAR1 [9, 33], which is equivalent to the human p.P193A mutation. For cross-reference with the human AGS mutation, we arbitrarily refer to the mouse K948N mutation as K999N mutation and this mutant mouse as the AGS ADAR1^K999N^ mouse. The P195A mutant mouse was referred to as the ADAR1^P193A^ mouse.

The p.P193A ADAR1 mutation is the most common of 28 identified ADAR1 mutations in AGS patients and the K999N ADAR1 mutation is the 3rd most common [9]. However, both heterozygous and homozygous ADAR1^P193A^ mice showed no differences from wild type mice in either morphologic or biochemical studies (data not included). Given that the P193 A mutation occurs in conjunction with other AGS mutations in symptomatic AGS patients [9, 33], and that our P193 homozygotes do not show abnormality, we deferred detailed analysis of these mice.

**Fig. 1:**
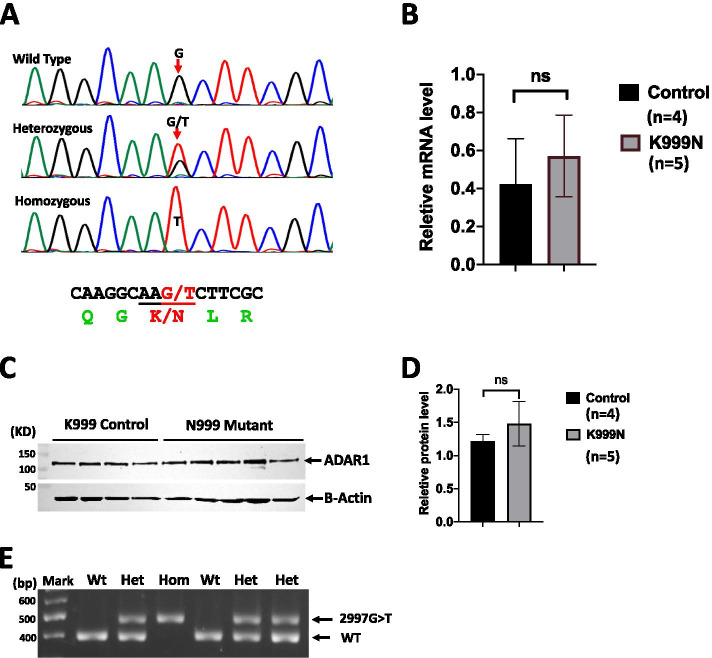
ADAR1 K999N mouse preparation. **A** Using CRISPR/Cas9 technology, a single nucleic guanosine (G) to thymidine (T) mutation was introduced into the mouse genome that codes the K948N mutation in mice, equivalent to the K999N mutation found in AGS patients. The genome G > T replacement was confirmed by Sanger sequencing in the founder and progenies*.***B** This mutation does not alter the level of gene transcription or ADAR1 protein expression in the brain, as shown by real-time RT-PCR (*n* = 5, *p* = 0.905) and **C** Western blot analysis. ADAR1 protein levels in 4 wt and 5 mutant mouse samples are shown. **D** PCR genotyping was optimized to distinguish the wt and mutant alleles. The specific amplifications for G > T mutant and wt alleles are as indicated. The Wilcoxon rank-sum test was used to determine the statistical difference for the analysis in **B** and **D**

For the ADAR1^K999 N^ mouse strain, the c.2844 G > T mutation was confirmed in the founder and the following generations via Sanger sequencing (Fig. 1A). We also confirmed that ADAR1 mRNA was expressed at the same levels in the brains of mutant and wild type (wt) mice (Fig. 1B). Additionally, ADAR1 protein levels in the brain, measured using Western blot analysis, were comparable between the mutant and wt controls (Fig. 1C, D). Hence, this specific AGS mutation was successfully introduced to the mouse genome and its expression is comparable with the wt control. To maintain this strain, an optimized PCR genotyping assay was established that distinguishes the c.2844 G > T mutant allele from the wt (Fig. 1E).

In this report, we focus on the functional and phenotypic effects of the p.K999N mutant in ADAR1^K999N^ mice. Progeny of ADAR1^K999N^ heterozygotes followed a normal Mendelian distribution for wild type, heterozygous, and homozygous mutant genotypes, and no obvious abnormality was observed, indicating that embryonic and perinatal development was not affected by the ADAR1^K999N^ mutation. The homozygous mice grew to adulthood and were generally healthy without manifesting spasticity or dystonia. They were fertile with litter size comparable to wild type mice.

### Gross and extracephalic phenotype of mutant mice

Patients with AGS have been reported to have a slower post-natal growth rate [37], and intrauterine growth retardation has also been reported [9]. Other non-CNS symptoms include a systemic lupus erythematosus (SLE)-like phenotype, including chilblain lesions affecting the fingers, toes, and ears [3, 38]. Hepatosplenomegaly and liver inflammation with elevated liver function enzymes have been described [3, 38, 39].

**Fig. 2:**
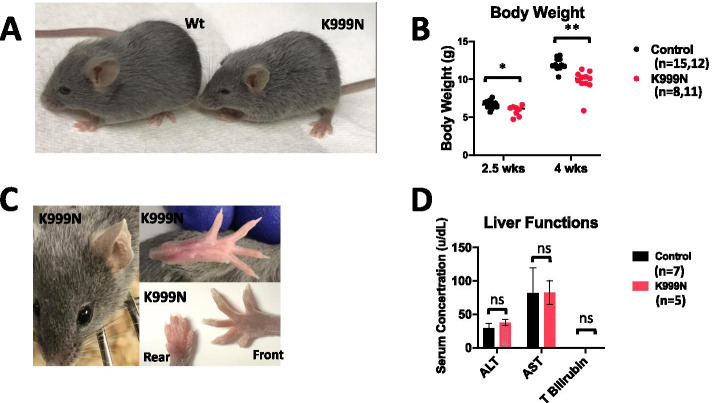
Phenotypic changes in ADAR1^K999N^ mice. **A** As shown at 3.5 weeks of age, the K999N mice are smaller with no gross structural abnormalities. **B** The difference in body weight between wild type and mutant mice increases over time, *n* = 15, 12 for control at 2.5 and 4 weeks, *n* = 8, 11 for K999N group at 2.5 and 4 weeks. *p* = 0.004 for 2.5 weeks and *p* = 0.001 for 4 weeks. **C** Although AGS patients can exhibit chilblain-like dermatologic manifestations in the ear, hands, and feet, no gross changes in mouse paws were evident in mutant mice. **D** Liver enzyme levels in control (*n* = 5, black) versus mutant (*n* = 7, red) mice for ALT, AST, and level for T. Bilirubin were not different, *p* = 0.066 (ALT), 0.404 (AST), and 1.0 (T. Bilirubin). The Wilcoxon rank-sum test was used to determine the statistical difference for the analysis in **B** and **D**

The most notable gross phenotype of ADAR1^K999N^ mice was smaller size (Fig. 2A) and body weight (Fig. 2B) that continued to decline with age. As described in patients, the relative growth retardation of these mice compared to controls increased over time [37].

**Fig. 3:**
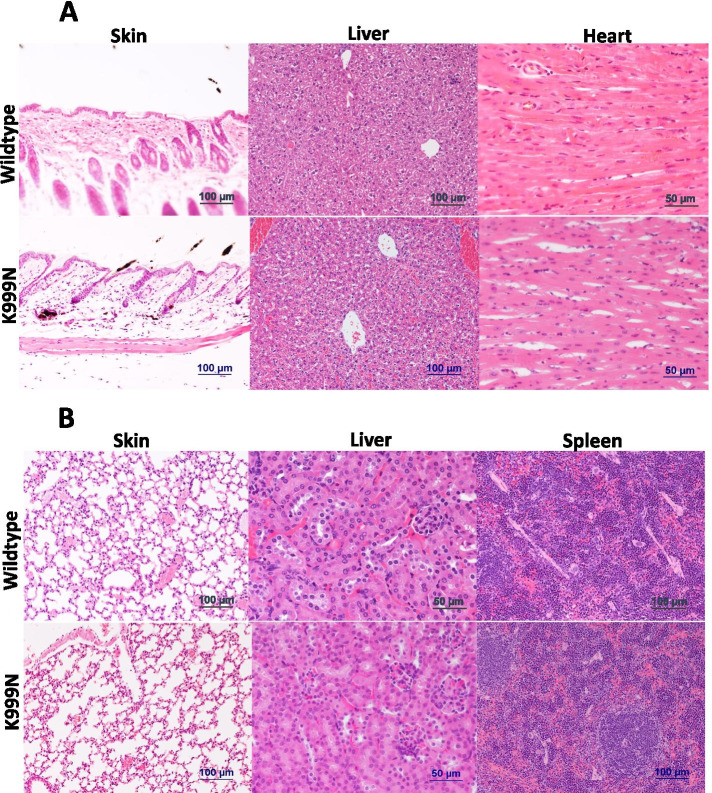
Morphology of extracephalic organs. **A** Hematoxylin/eosin-stained tissue sections of the skin, liver, and heart, and **B** hematoxylin/eosin-stained tissue sections of the lung, kidney, and spleen tissues of wild type control and ADAR1^K999N^ mutant mice were shown. No histological difference was observed in ADAR1^K999N^ organs from wild type controls

Gross evidence of a lupus-like syndrome and of organ inflammation was absent. We observed no obvious skin lesions compatible with chilblains in the ears and toes (Fig. 2C). There was no significant difference from wild type mice in AST, ALT, or bilirubin indicative of liver damage in the ADAR1^K999N^ mice (Fig. 2D). The H&E sections of the skin, liver, heart, lung, liver, and spleen were microscopically normal and free of inflammation (Fig. 3A, B).

### RNA editing activity in ADAR1^K999N^ mutant mice

For context in studying aberrant cellular RNA processes in the ADAR1^K999N^ mice, we evaluated indicators of the mutant ADAR1 function in vivo. The ability of ADAR1 to catalyze adenosine to inosine substitution in RNA is required in order for ADAR1 to carries out its RNA editing functions. Of the 8 ADAR1 mutants originally reported in AGS, 7 (including K999) localize to the catalytic domain [33]. Based on the crystal structure of the homologue protein ADAR2 bound to RNA, K999 was situated near the protein-RNA interface such that K999N might alter RNA binding [40].

ADAR1 sequences are highly conserved between human and mouse in their catalytic domains as shown by the sequence alignment (Supplemental Fig 2). In order to confirm that the K948N mutation in the mouse would be structurally analogous to the AGS K999N patient mutation, we compared their protein structure using the modeling software Modeller. This analysis confirmed a high structural similarity between these two proteins (Supplemental Fig 3A). In addition, our analysis showed that the K999 (K948 in mouse) forms a strong salt bridge with the E1014 residue (E963 in mouse) (Supplemental Fig 3B). By disrupting this salt bridge, the K999N mutation likely causes a conformational change of ADAR1 that could impact catalytic or binding function.

**Fig. 4:**
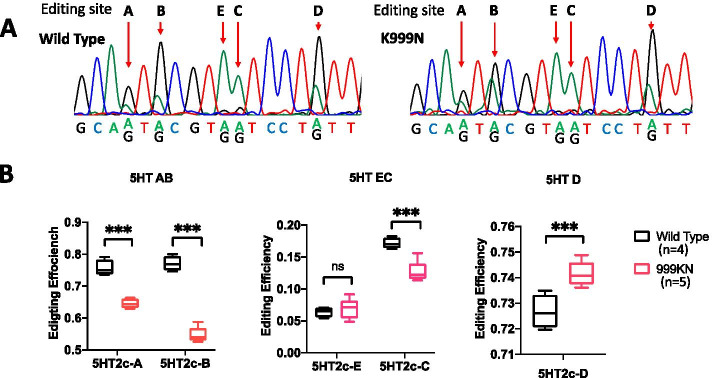
ADAR1^K999N^ mutation causes RNA editing changes on neuron 5HTR2c mRNA substrate. The RNA editing activities of the K999N mutant protein in the mouse brain on 5HTR2c mRNAs were assessed by sequencing analysis. RNAs from whole brain tissues were amplified by PCR with specific primers for 5HTR2c following reverse transcription, and the PCR products were subject to Sanger sequencing analysis. **A** The editing levels of 5HTR2c mRNA at A, B, C, D, and E sites in ADAR1^K999N^ and wild type control mice were shown by the representative chromatograms of brain RNA Sanger sequencing. The editing sites are marked by the arrows. **B** The editing level on each editing site was compared between ADAR1 K999N mutant (*n* = 5) and wild type (*n* = 4) mice. Editing on A, B, and C sites in ADAR1^K999N^ mice was significantly decreased. However, editing on the D site was increased by the mutation. *p* < 0.05 for A, B, C and D sites. Editing at the E site occurred at baseline low efficiencies, with no differences in editing efficiency between the ADAR1^K999N^ mice and WT controls. The Wilcoxon rank-sum test was used to determine the statistical difference between ADAR1^K999N^ mice and WT controls

We compared the editing levels in RNA transcripts at sites targeted by ADAR1 in ADAR1^K999N^ and control mouse brains to determine whether K999N mutation caused changes in ADAR1 editing activities. RNAs used for ADAR1^K999N^ editing activity analysis were isolated from total brains, reversely transcribed and amplified by PCR, and then subjected to Sanger sequencing analysis. Editing efficiency at each site was determined by the relative quantities of the *G* (adenosine is edited to inosine which is read as guanosine) versus *A* (unedited, consistent with genomic code) peaks on the sequencing chromatograms (Fig. 4A). Quantification of results showed that differences in editing were found in the 5-Hydroxytryptamine Receptor 2C (5-HTR2c) mRNA in the mutant mice. Editing activity at the A, B, and C sites within the transcript was significantly decreased while editing at the D site was increased (Fig. 4B). In contrast, editing at ADAR1 target sites in Glutamate Ionotropic Receptor AMPA Type Subunit 2 and 3 (GRIA2 and 3) was unchanged from wild type (Supplemental Fig 4A-F). ADAR1^K999N^ mutation was therefore associated with decreased, increased, or unchanged levels of editing efficiency depending on different neuronal RNA substrates and different editing sites, supportive of an effect of the mutation on cellular RNA modification.

### ADAR1^K999N^ mutation effects on inflammatory cytokine levels in the brain

The effect of the K999N mutation in the mouse on selective editing of ADAR1-targeted brain mRNA sites raised the question of whether the editing defect could trigger the induction of inflammatory cytokines and interferon pathway gene expression in the brain.

**Fig. 5:**
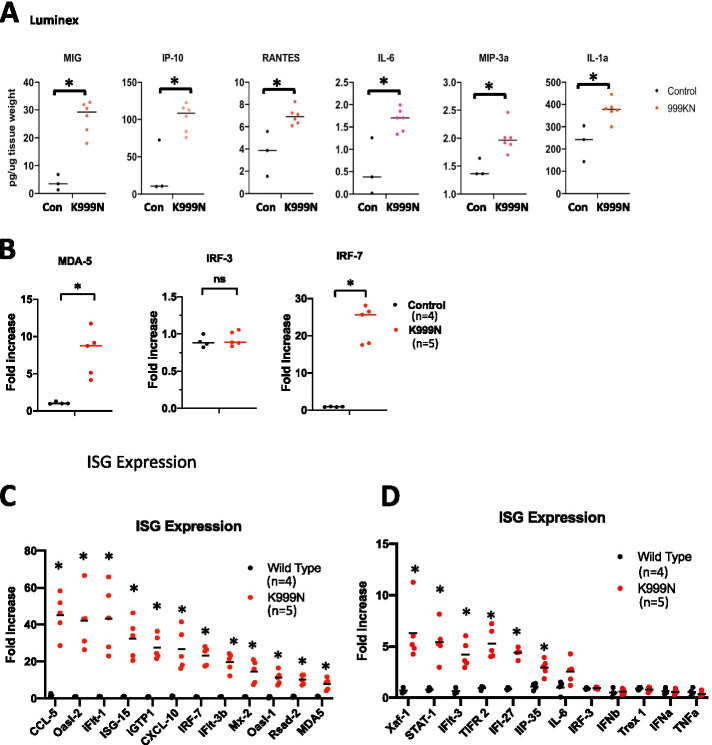
ADAR1^K999N^ mutation results in increased ISG expression in the brain. **A** Inflammatory cytokines and chemokines were measured in the brain protein extracts using Luminex assays. The cytokine and chemokine levels were significantly increased in ADAR1^K999N^ mice. *n* = 3 (wt, black dots) and 5 (K999N mutant, red dots), *p* = 0.024 (MIG), 0.020 (IP-10), 0.024 (RANTES), 0.020 (IL-6), 0.019(MIP-3a), and 0.048 (IL-1a), with statistical analyzed using Wilcoxon rank-sum test. **B** MDA-5, IRF3, and IRF5 expression in brain tissues of wild type and ADAR1^K999N^ mice was quantified using a real-time PCR approach. MDA-5 expression is significantly increased in ADAR1 ^K999N^ mice, *n* = 4 (wild type), *n* = 5 (ADAR1^K999N^), *p < 0.05. While IRF-3 levels do not show a difference between the two groups, IRF-7 expression is more than 25 fold increased in ADAR1^K999N^ mouse brain with comparison to the controls, *n* = 4 (wild type), *n* = 5 (ADAR1^K999N^), *p < 0.05, as analyzed using the Wilcoxon rank-sum test. **C**, **D** Interferon-stimulated gene (ISG) expression in brain tissues of wild type and ADAR1^K999N^ mice was quantified using a real-time PCR panel covering 24 ISGs*.* Within this panel, mRNA levels of 18 ISGs are significantly increased in ADAR1^K999N^ mice, with 20- to 70-fold increase of the top 7 ISGs including CCL-5, Oasl-2, ifit-1, ISG-15, iGTP1, CXCL-10, and IRF-7. *n* = 4 (wild type), *n* = 5 (ADAR1^K999N^), *p < 0.05, with statistical analyzed using the Wilcoxon rank-sum test. The gene expression levels in **B**–**D** were calculated using the ΔΔt method with reference to the average of GAPDH and HRTP. Increases were expressed as fold changes relevant to levels in wild type mice

We measured whether inflammatory chemokines and cytokines were increased throughout the brain in mutant compared to wild type animals. We prepared protein lysates from homogenized mouse brains and chemokine/cytokine levels in the brain lysates were measured using a Luminex Assay for 45 chemo/cytokines. The results showed significantly elevated levels of inflammatory cytokines, including IL-6, IL-1, IL-17, MIG (CXCL9), IP-10 (CXCL10), RANTES (CCL5), MIP-3a, Eotaxin, KC, and TARC (Supplement Fig 6), indicating that the ADAR1^K999N^ mutation led to an inflammatory gene expression in the brain. Notably, IFN-regulated chemokines MIG, IP-10, and RANTES, which were used for patient CSF analysis [9, 41], are the most significantly upregulated cytokines on Luminex assay (Fig. 5A).

### ADAR1^K999N^ mutation effects on gene expression of ISGs in the brain

The molecular mechanism that links the AGS-associated mutations to brain inflammatory injuries remains to be determined. We and others have shown that deficiency of ADAR1 causes cellular RNAs to activate innate immunity by enabling their detection by the RNA receptor MDA-5, thereby triggering the cytosolic RNA sensing signaling pathway through interferon regulatory factors (IRFs), resulting in elevated ISG expressions. Excessive ISG expression is central to AGS as an inflammatory interferonopathy [3, 8, 12]. This ADAR1^K999N^ mouse model enabled us to determine whether an ADAR1 mutation that alters neuronal RNA editing patterns (Fig. 4) activates ISG expression in the brain of ADAR1^K999N^ mice. Total RNA was isolated via whole brain homogenization, and expression of a 24-gene panel of IFN and ISGs was measured via real-time RT-PCR. Interestingly, the expression level of MDA-5 was dramatically increased in mutant mice. Additionally, the expression of IRF 7, the key transcription factor for IFN signaling pathway, was more than 20-fold upregulated, while IRF3’s expression was not affected (Fig. 5B). Although IFN-α and IFN-β gene expression was comparable between wild type and mutant brains, there was nonetheless a robust increase in ISG expression profile in the ADAR1^K999N^ mice (Fig. 5C, D). Transcripts for CCL-5, Oasl-2, IFIT-1, ISG-15, iGTP1, CXCL-10, IRF-7, and iFIT-3b were increased more than 20-fold, while in total 17 of the assayed ISGs were expressed at significantly higher levels in the ADAR1^K999N^ brain than in the controls (Fig. 5C, D). Intriguingly, four of the genes included in this panel, IFI-27, IFIT-1, ISG-15, and RSAD2, are among the six biomarkers measured in peripheral blood to calculate the interferon score used as part of AGS patient evaluation [3]. The increased expression of these genes in the ADAR1^K999N^ mouse brain is analogous to the interferonopathy of AGS patients [9, 33], showing innate immune signaling pathway activation in the brain tissues.

### ISG expression pattern and cell types in ADAR1^K999N^ mouse brains

**Fig. 6:**
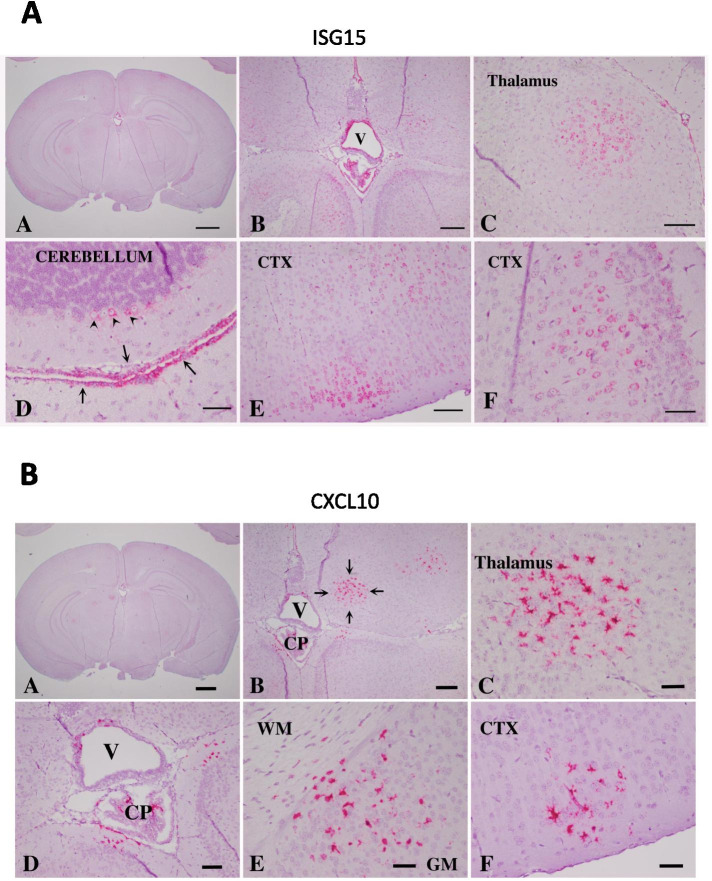
Distribution of ISG expression in ADAR1^K999N^ mouse brain by RNA in situ hybridization (ISH). ISG expression on formalin-fixed paraffin-embedded (FFPE) sections of ADAR1^K999N^ mice were detected by ISH for ISG15 and CXCL10 counterstaining with hematoxylin. **A** ISH for ISG15. (A) Low power coronal section shows multiple foci of hybridization (red). Bar = 1 mm. (B) Medium power allows resolution of staining in the choroid plexus and ependyma surrounding ventricle (V). Bar = 200 μm. (C) High power of the thalamus showing hybridization within a discrete focus of neurons. Bar = 100 μm. (D) High power of the cerebellum shows hybridization in Purkinje neurons (arrowheads) and pia matter (arrows). Bar = 100 μm. (E) High power of cortical gray matter shows hybridization in a subpopulation of neurons. Bar = 100 μm. (F) The highest power of cortical gray matter shows hybridization in neuronal cytoplasm. Bar = 50 μm. **B** ISH for CXCL10 (red). (A) Low power coronal section shows multiple foci of hybridization (red). Bar = 1 mm. (B) Medium power allows resolution of staining in the choroid plexus (CP) and ependyma surrounding ventricle (V) and focus on the thalamus (between arrows). Bar = 200 μm. (C) High power of the thalamus shows a circular focus of hybridization consisting of cells with blunted processes amidst normal appearing neurons. Bar = 100 μm. (D) High power of the ventricle shows hybridization in ependyma and choroid plexus cells. Bar = 100 μm. (E) High power of cortical gray matter (GM) and white matter (WM) shows hybridization in microglia with blunted processes in both regions. Bar = 100 μm. (F) High power of cortical gray matter shows a solitary focus of hybridization in microglia with blunted processes. Bar = 100 μm

**Fig. 7:**
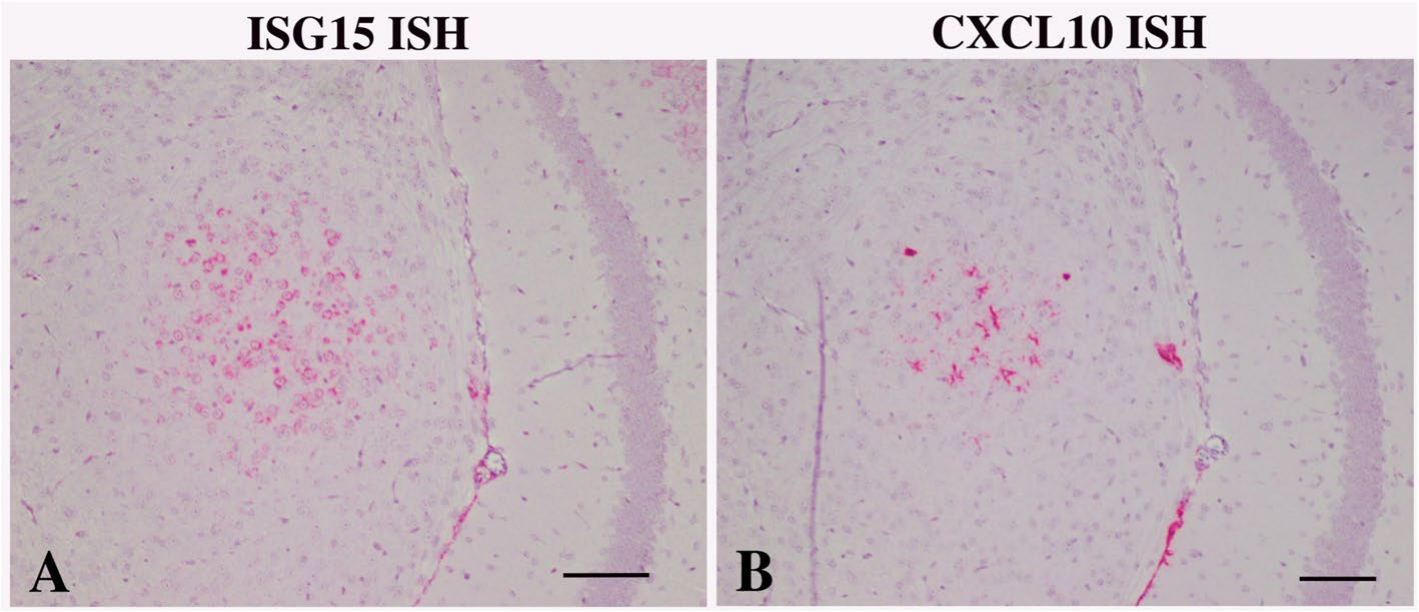
Cell type-specific expression of different ISGs. Successive FFPE sections of ADAR1K999N mice following in situ hybridization for ISG15 (**A**) or CXCL10 (**B**) and counterstaining with hematoxylin. **A** A discrete focus on neurons hybridizing for ISG15 (**A**) overlaps with a similarly constrained focus of CXCL10 hybridized microglia in the successive section (**B**). Bar = 100 μm

In order to ascertain whether the increased interferon pathway signaling in mutant mice was global or topographically restricted, RNA in situ hybridization (ISH) was performed. We chose to analyze ISG-15, one of the interferon score genes upregulated in AGS CSF and blood [42], and CXCL10 that has been detected in glial cells of postmortem AGS patient brain [41]. ISH detected ISG-15 expression in widely distributed cortical and subcortical neurons in addition to choroid plexus and ependyma (Fig. 6A and Supplemental Fig 7). Expression in neurons appeared in unusual neural clusters that were neither distributed symmetrically nor distributed in neuroanatomically related regions. ISH detected CXCL10 expression in widely distributed clusters of cells morphologically consistent with microglia (Fig. 6B and Supplemental Fig 8). As with ISG-15, the clusters of CXCL10-positive microglia were neither distributed symmetrically nor distributed in neurophysiologically functional domains. Sequential brain sections were used to compare the relationship between regions expressing the two ISG genes. Clusters of ISG-15-positive neurons overlapped with CXCL10-positive microglia (Fig. 7). Comparison to GFAP- and IBA1-immunostained sections confirmed the absence of astrocytosis and microgliosis in regions expressing ISG15 or CXCL10, nor elsewhere in the brain (Supplemental Fig 9). Routine histopathological analysis of the ADAR1^K999N^ mouse brain did not detect cortical or subcortical pathology with H&E staining, and Von Kossa and Luxol Fast Blue staining confirmed the absence of mineralization or white matter pathology (Supplemental Fig 10).

## Discussion

Here, we provide in vivo evidence that an AGS-associated mutation in ADAR1 is sufficient to activate the IFN signaling pathway in the brain, a central feature of AGS pathology. The work was enabled by a successful generation of the mouse model carrying the ADAR1^K999N^ mutation, the first animal model of a specific AGS-associated mutation with robust ISG expression in the brain. The expression of different ISGs in cortical and subcortical neurons and microglia exhibits a patchy pattern, including the basal ganglia areas. We also show that altered RNA editing occurs in the mutant neuronal cells, supporting that dysregulated processing of nucleic acids plays a role in AGS pathogenesis.

Multiple animal models have knocked out or mutated AGS-linked genes. However, these models have been limited in their ability to explain the central nervous system pathology of AGS. TREX1 is the major 3′ → 5′ DNA exonuclease in mammalian cells that removes the 3′ overhangs for DNA repair [43, 44]. Knockout of TREX1 gene [16, 17] or introducing a missense mutation in its catalytic domain [15] resulted in innate immune activation and inflammatory myocarditis [14] that promoted the early death of mutant mice at an average of 6 months of age. A modest increase of CXCL10 expression in the brain was also reported in a global Trex1 knockout mouse model [45]. Peripheral autoimmunity was linked to loss of the gene in dendritic cells, and specific deletion of Trex1 in neuroglial cells led to a minimal to a modest increase of 3 ISGs, while there was no measurable effect on neurons in the brain [18]. Whether a specific AGS mutation in an animal could lead to brain IFN pathway activation is not known.

Mutations in the three subunits of RNase H2 (RNase H2a, 2b, and 2c) comprise more than 50% of all genetic alterations seen in AGS patients [2, 11, 46]. The most common missense mutation found in AGS, *Rnaseh2b*^*A174T/A174T*^, caused an increase in ISG’s in the heart and kidney but not in the brain [47]. In another study, knock-in mice with a mutated RNase H2 lacking catalytic activity did not have a brain phenotype although isolated fibroblasts displayed augmented interferon pathway activation [48]. Brain-specific deletion of RNase H2 did not lead to inflammatory response or a ISG signature in the brain tissue, despite the fact that ISG transcripts were elevated in isolated astrocytes from these mice [49]. SAMHD1-deficient mice do not develop any detectable pathology or autoimmunity [50]. No organs from SAMHD1-deficient mice, including the brain, have demonstrated any inflammatory changes [50].

We had attempted to target all of the 8 missense mutations in the ADAR1 gene that were first reported [33], but failed to obtain a mouse carrying any of the desired mutations in multiple trials. We confirmed the highly efficient excision/repair activities of the designed sgRNAs at the targeted sites in early embryonic cells (data not shown), but high death rates were encountered in embryos targeted by ADAR1 sgRNAs. Eventually, two mutations were successfully introduced into the mouse genome after screening more than 100 living pups. ADAR1 gene disruption is known to be embryonic lethal [20, 21, 26, 27]. Our study proved the difficulty in generating mice with mutations at ADAR1 gene loci.

AGS-associated mutations have significant heterogeneity in clinical manifestations [2, 3, 51]. Some of them are associated with severe brain injuries, and some are asymptomatic. The significant heterogeneity of clinical manifestations even occurs among affected members of the same family [9, 23, 24]. Conceivably, ADAR1 K999N and other associated mutations are necessary but not sufficient for all disease manifestations, and the disease worsens in some patients following an environmental or infectious trigger [9]. Mice for this study were housed in a controlled specific pathogen-free facility, and the relative paucity of environmental stimuli may partially explain the milder phenotypes observed in our model. Nevertheless, spontaneous IFN pathway activation occurred in ADAR1^K999N^ mice, in both neurons and microglia that produce different ISGs. Furthermore, we found that both cortical and subcortical neurons express ISG-15 and not CXCL10 while microglia express CXCL10 and not ISG15. The distribution of cells showing increased ISG expression is also unusual, neither symmetric nor neuroanatomically logical. Despite elevated expression of ISGs, there is no evident neuroinflammation either astrogliosis or microgliosis. Together, these findings suggest that the mutant murine model represents the initial portion of the human disease AGS, but may be missing a second hit such as an environmental stress (e.g., trauma, infection, stroke, etc.) that would solicit neuroinflammation. This would be compatible with the heterogeneity of AGS manifestations and time course within affected family members. It is not clear why only certain cells were producing cytokines and why they did not elicit noticeable inflammation. In the absence of additional stressors, uncoordinated expression of ISGs may not translate into pathological inflammation [52–54].

The AGS phenotype is broad, including both motor defects and spasticity, but also seizures, encephalopathy, and intellectual impairment. It was of interest that the topographic distribution of upregulated ISG RNA was similarly broad in our in situ RNA analysis, yet did not strictly map to functionally associated regions. Further study is needed to investigate the source of this variation. We do note that areas associated with AGS pathology, notably basal ganglion, were included among ISG-positive areas.

Excessive IFN signaling underlies many autoimmune diseases including inflammatory encephalopathies. However, studies on the pathogenic development of brain diseases are challenging because of the limited accessibility of brain tissue, especially during development. The ADAR1^K999N^ mice may be useful in clarifying the effects of aberrantly activated inflammatory signals both in AGS and other neuroinflammatory diseases. ADAR1 knockout in Schwann cells and spinal neurons upregulated ISGs and blocked oligodendrocyte differentiation in an ISG (IFIT-1)-dependent manner [30]. In the case of ASG, should a second hit model (e.g., viral challenge) unmask the full AGS phenotype in our mutant mice, it will be of interest to see whether this can be prevented by a specific ISG blockade.

## Conclusions

A mouse model, the first animal model of a specific AGS-associated mutation capable of causing robust ISG expression in the brain, was successfully established. This mutation is equivalent to human ADAR1 p.K999N found in AGS patients. We demonstrated that this single nucleotide mutation is able to activate the IFN pathway in the brain. Neurons and microglia expressed different ISGs. This mutant mouse replicated aspects of the brain interferonopathy of AGS. The absence of the brain morphological abnormalities typical of AGS in this mouse model indicated that additional “hit(s)” might be necessary for the development of the full clinical AGS phenotype. This mutant mouse presents a robust tool for the investigation of AGS and neuroinflammatory diseases including the modeling of potential “second hits” that enable severe phenotypes of clinically variable diseases.

## Supplementary Information

**Additional file 1: Figure S1.** Illustration of the mutation sites in ADAR1 protein.The mutation sites and the relative positions in ADAR1 protein of the 8 originally reported missense mutations are shown. All these mutations were designed to be introduced to mouse genome. The successful sites are indicated by arrows above the protein (blue), other 6 below the protein (black). **Figure S2.** Sequence alignment of mouse and human ADAR1 protein sequences. Mouse and human ADAR1 protein sequences were compared using Promals3D software (http://prodata.swmed.edu/promals3d/promals3d.php). The K948 in mouse is equivalent to K999 in human, highlighted in red. Sequences of mouse ADAR1, Q99MU3 and human, P55265 in UniPro protein database were used for alignment. **Figure S3**. Human and mouse ADAR1 structure comparison. 3A, the highly conserved protein structure between mouse and human in the catalytic domain flanking the K999N mutation site is shown by the protein modeling, showing a RMSD of 1.06 Å in the protein main chains, supportive of similar structure. 3B, Protein structure analysis predicted a salt bridge (green lines) between K948 (K999 in human) and E964 (E1004 in human), which stabilize the loop in which K948 and E964 localize in the catalytic domain. **Figure S4. **The RNA editing activities of the K999N mutant protein in mouse brain. 4A, C and E, RNAs from whole brain tissues were amplified by RT-PCR for GRIA2, GRIA3, GRIK1 mRNAs. and the PCR products were subject to Sanger sequencing analysis. The representative chromatograms for the sequences flanking the edited adenosines at +60 site and R/G site in GirA2 mRNA, R/G site in GirA2 mRNA, and Q/R site in Girk1 mRNA are shown. 4B, D and F, The RNA editing activities as measured by the relative ratio of the G peak (edited) over the total A (unedited) and G peak areas at each editing site of the ADAR1 K999N mutant (n=5) vs wild type (n=4) are compared, and no statistical difference was observed as analyzed using Wilcoxon rank-sum test. **Figure S5**. RNA editing at the ADAR2-targeted GRIA-2 Q/R site. The efficiency of RNA editing on the mRNA coding GRIA-2 subunit at the Q/R site was assayed. Total RNAs were isolated from whole brain tissues of wt and ADAR1^K999N^ mice, then RT-PCR was performed, followed by Sanger sequencing. The chromophotographs of Sanger sequencing flanking the Q/R site, known edited by ADAR2, are shown. The genomic coded adenosine was almost completely converted to inosine, which is read as guanosine. The editing site is pointed by the arrow. **Figure S6.** Luminex assays of the mouse brain protein extracts. Protein extracts from wild type and ADAR1 K999N mice were subject to Luminex assays for cytokine and chemokine levels. The panel profiles 45 cytokines/chemokines. Increases in measured concentration of each cytokine in the ADAR1^K999N^ mice were calculated in terms of fold changes relative to wild type mice. *n*=3 (wild type), *n*=6 (K999N), * *P*<0.05 with statistical analyzed using Wilcoxon rank-sum test. **Figure S7.** ISH for ISG-15 on mouse brain sections. In situ hybridization for ISG-15 transcripts in paraffin sections from wildtype (A-C) and mutant (D-F) mouse brains. A&D, Low power coronal section of whole mouse brain demonstrates no staining in wildtype (A) and multiple foci of hybridization (red staining) in mutant brain (D). B&E, Medium power of periventricular region demonstrates no staining in wildtype (B) and abundant staining in ependyma and parenchymal regions in mutant brain (E). C&F, Medium power of neocortex demonstrates no staining in wildtype (C) and abundant staining in ependyma and parenchymal regions in mutant brain (F). Bar = 1 mm in A&D; =100 microns in B, C, E &F. **Figure S8. **ISH for CXCL10 on mouse brain sections. In situ hybridization for CXCL10 transcripts in paraffin sections from wildtype (A-C) and mutant (D-F) mouse brains. A&D, Low power coronal section of whole mouse brain demonstrates no staining in wildtype (**A**) and multiple foci of hybridization (red staining) in mutant brain (D). B&E, Medium power of periventricular region demonstrates no staining in wildtype (B) and abundant staining in ependyma and parenchymal regions in mutant brain (E). C&F, Medium power of neocortex demonstrates no staining in wildtype (C) and abundant staining in ependyma and parenchymal regions in mutant brain (F). Bar = 1 mm in A&D; =100 microns in B, C, E &F. **Figure S9. **Immunohistochemically stained for GFAP and IBA1. FFPE sections of hippocampus of wildtype (A&C) and ADAR1^K999N^ (B&D) mice immunohistochemically stained for GFAP (A&B) and IBA1 (C&D) show no difference in astrocytosis or microgliosis. Counterstained with hematoxylin. Bar = 200 microns. **Figure S10. **H-E, Luxol Fast Blue (LFB) and Von Kossa staining on brain sections. FFPE sections of mutant (A&B) mice and wildtype (C&D) mice following Von Kossa (A&C) and LFB (B&D) staining. There is no evidence of calcification on Von Kossa staining, nor evidence of demyelination on LFB staining. Bar = 1mm. **Figure S11. **Original image of Western Blot for Fig 1 panel C. Western blot was stained with ADAR1 and beta-Actin antibodies on parallel blots with same quantity of protein loading.

## Data Availability

All data was presented in the main manuscript or additional supporting files.

## Abbreviations

AGS: Aicardi-Goutières syndrome
SLE: Systemic lupus erythematosus
ADAR1: Adenosine Deaminase Acting on RNA 1
5-HTR2c: 5-Hydroxytryptamine receptor 2C
GRIA: Glutamate Ionotropic Receptor AMPA Type Subunit
IFN: Interferon
ISG: Interferon-stimulated gene
CXCL: C-X-C motif chemokine ligand
IFIT-1: Interferon-induced protein with tetratricopeptide repeats 1
FFPE: Formalin-fixed paraffin-embedded
ISH: In situ hybridization
LFB: Luxol Fast Blue
